# The Influence of Roughness and Pyrethroid Formulations on Bed Bug (*Cimex lectularius* L.) Resting Preferences

**DOI:** 10.3390/insects6020455

**Published:** 2015-05-12

**Authors:** Benjamin A. Hottel, Roberto M. Pereira, Philip G. Koehler

**Affiliations:** Department of Entomology and Nematology, University of Florida, 1881 Natural Area Drive, Gainesville, FL 32611, USA; E-Mails: rpereira@ufl.edu (R.M.P.); pgk@ufl.edu (P.G.K.)

**Keywords:** *Cimex lectularius*, roughness preference, pyrethroid avoidance, behavioral resistances

## Abstract

Two-choice tests were conducted to examine the effect of surface roughness on the resting preference of bed bugs, *Cimex lectularius* L., on copper, basswood, and acrylic materials. The influence of pyrethroid formulation applications on resting preferences was also evaluated. Bed bugs were given the choice of resting between two sanded halves of each material tested. One half was sanded with a P60 grit sandpaper and the other with a less rough P600 grit sandpaper. A significantly higher proportion of bed bugs chose to rest on the rougher P60 grit sanded half of all materials tested. Pyrethroid applications were made to either the P60 grit half or both halves of acrylic arenas and resting preferences were again assessed. Behavioral responses of bed bugs to pyrethroid formulation applications varied depending on the bed bug strain used and the formulation applied. Bed bugs would still rest on the P60 grit half when Suspend SC formulation (0.06% deltamethrin) was applied; however, an avoidance response was observed from a bed bug strain susceptible to D-Force aerosol formulations (0.06% deltamethrin). The avoidance behavior is likely attributed to one, more than one, or even an interaction of multiple spray constituents and not the active ingredient.

## 1. Introduction

The common bed bug, *Cimex lectularius* L. (Hemiptera: Cimicidae), is a very cryptic species found hiding in a variety of locations while they are not feeding on their human hosts [1]. Locations include: crevices and corners of wooden bed boards, behind loose wallpaper, in nail holes and cracks in the walls, behind pictures, conduits, light switches, wall panels, baseboards, door and window frames, floor crevices, and furniture and mattresses. It may be no surprise that many of these locations are common areas that pest control operators treat with insecticides to eliminate a current bed bug infestation [2]. While this list holds true in many bed bug infestations, there are always exceptions, which make it important to understand why bed bugs harbor in certain locations over others.

A handful of studies have investigated bed bug harborage preferences over the past 100 years. Investigations concerning the harborage preferences of bed bugs have recently focused on the chemical nature of bed bug aggregation. Items that were previously harbored upon by bed bugs were found to be more attractive than plain control options [3,4,5]. These findings led to the conclusion that bed bugs have an airborne and contact aggregation pheromone that may help them find previously established harborages.

The influence of other factors on the harborage preference behavior of bed bugs has not been examined for quite some time. Hase [6], cited by Usinger [1], stated that bed bugs prefer to harbor in locations that are dry (*i.e.*, negative hydrotaxis), rough, and provide at least some partial darkness (*i.e.*, negative phototaxis). Latter studies supported much of the work done by Hase [6]. Experiments performed by Rivnay [7] examined many different taxes related to bed bug harborage behavior including negative phototaxis, negative hydrotaxis, and others. In one study, he found that bed bugs preferred to be in contact with an object as opposed to out in the open (*i.e.*, thigmotaxis) and that this thigmotactic behavior was stronger than their negative phototaxis. Rivnay [7] also observed that bed bugs would harbor around a piece of blotting paper as opposed to a nail and argued that this was evidence for their preference to harboring against a rough verses smooth surface. Some observational studies performed by Aboul-Nasr and Erakey [8] examined the roughness preference behavior of bed bugs on a variety of materials but these studies lacked the details needed to discern what was actually being quantified during the experiments.

Besides the numerous studies that have examined the biochemical and physiological resistance mechanisms of bed bugs to pyrethroids [9,10,11,12], one study has investigated the effects of behavior resistance in relation to harborage preferences [13]. These authors observed the effects of 0.06% deltamethrin (Suspend SC, Bayer Environmental Science, Research Triangle Park, NC, USA) applications on negative phototaxis and harborages with aggregation pheromones. Repellency to harborages treated with pyrethroids was observed in bed bugs when negative phototaxis was used as the primary factor to attract bed bugs to the harborage; however, no repellency was observed when bed bugs were presented with harborages containing aggregation pheromones and pyrethroid formulation applications. No experiments yet have examined other harborage characteristics for behavioral responses to pyrethroid applications.

In our study we examined the roughness resting preference behavior of bed bugs on various materials. We also investigated the effects of pyrethroid applications on roughness preference of bed bugs.

## 2. Materials and Methods

### 2.1. Insects

A lab strain of *C. lectularius* collected by Harold Harlan in 1973 from Ft. Dix, N.J. was used for all experiments. Another strain collected in Bradenton, FL in August 2013 was used on all acrylic experiments to compare potential strain differences in behavior. *Cimex lectularius* colonies were kept in 300 mL plastic jars. Folded filter paper was added as a substrate for the bed bugs to attach onto. Jar lids were modified to allow colonies to feed while being contained. A 7.5 cm diameter circle was cut out of the lids. A 90 μm nylon mesh was super glued and then hot glued over the tops of the lids. Bed bugs were fed on live chickens (University of Florida Institutional Animal Care and Use Committee approval 201303836_01) once every two weeks. Bed bugs were fed the same day they were used in any of the experiments. Colonies were kept at a temperature around 24 °C and exposed to natural light cycles (light: dark cycle at an average hour and standard error of 10.77 ± 0.05: 13.23 ± 0.05).

### 2.2. Surface Preparation

Materials representative of common surfaces found in households such as metal (copper sheets), wood (basswood), and plastic (acrylic sheet; Optix, Plaskolite, Inc., Columbus, OH, USA) were cut into 10 by 10 cm arenas. For each material, an abrasion treatment was applied to each half of the arena. Abrasion treatments were performed using a hand sander equipped with P60 or P600 grit sandpaper (3 M, Saint Paul, MN, USA). Sanding was done linearly in four different directions. Each direction was done 45° from the previous direction. Before an abrasion treatment was performed, basswood was sanded with P600 grit sand paper and then polished to a fine smooth finish using the back of the P600 grit sandpaper. Only acrylic and basswood arenas were reused. Acrylic arenas were cleaned with ethanol (EtOH) and basswood arenas were cleaned with a P80 grit belt sander.

### 2.3. Roughness Preference among Materials

Arenas for each material were split into two halves. Each half had an abrasion treatment of either a rough P60 grit sand paper or a smoother P600 grit sand paper. To confine the bed bugs within the treatment areas, the 5 cm diameter base of a 118 mL cup was removed and then hot glued upside down to the surface of each material. The upside down cup created a 7.5 cm diameter arena. The cup was placed so that the two treatment areas were equal.

A 50:50 ratio of male and female *C. lectularius* were used in this experiment. All experiments were run in a dark room and in absence of any human presence. Each individual was acclimated for one hour in the dark room. Individuals were placed in the center of each abrasion treatment combination. The abrasion treatment the bed bugs were resting on after one hour was recorded. Bed bugs that were observed on the midline between the P60 grit and P600 grit halves were not included in the analysis. Each abrasion treatment combination assay was replicated with 34 individual bed bugs. Bradenton strain bed bugs were only used on acrylic assays and 28 individuals were tested. Individuals alone were tested to reduce the potential effects of bed bug aggregation pheromones and thigmotaxis behaviors.

### 2.4. Roughness Preference with Pyrethroid Application

To test the influence of a pyrethroid application on roughness preference, assays were set up and conducted in the same manner as the roughness preference among materials, but only acrylic surfaces were used. Formulations of Suspend SC and D-Force Aerosol (FMC, Philidelphia, PA, USA) at a 0.06% deltamethrin concentrations were painted onto either the P60 grit sanded half or both the P60 and P600 grit half of the acrylic arena. Only one stroke of the paintbrush was made onto each half of the arena when using the D-Force formulation because the formulation had a tendency to spread past desired treatment areas. Treated arenas were dried for one to two hours before assays began. After recording the location of where the bed bugs were found, bed bugs were then removed from the assays and placed onto a 2.5 cm filter paper disc (GE Healthcare Life Sciences, Pittsburgh, PA, USA) inside a 30 mL plastic portion cups. In order to observe potential delayed mortality effects and recovery from initial knock down effects, mortality was assayed in all insecticide treatment assays after three days. Bed bugs were considered dead if they were unable to wright themselves after being turned onto their backs. Assays were replicated with individual bed bugs 28 times for both the Ft. Dix strain and the Bradenton strain on all treatment combinations.

### 2.5. Statistical Analysis

Data for all experiments were analyzed using an exact binomial test at alpha = 0.05. R statistical software (R Foundation, Wien, Austria; 2015) was used to analyze all data and create figures.

## 3. Results

**Figure 1:**
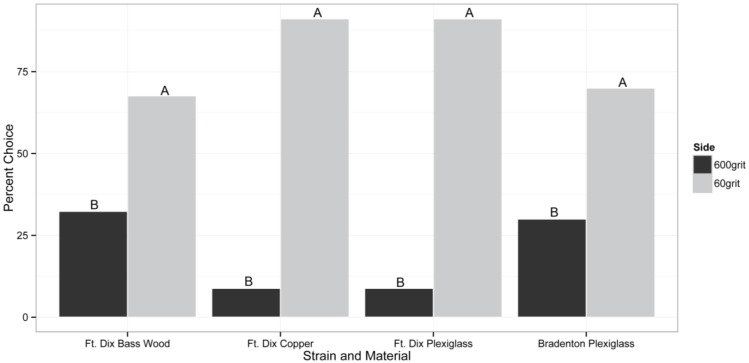
The roughness preference of individual adult bed bugs (50:50 sex ratio) given the choice between resting on the side of the assay with a surface sanded with a rough P60 grit or a smoother P600 grit piece of sandpaper. Both Ft. Dix and Bradenton strains were tested. Treatment sides with different letters represent statistically significant differences inferred from a binomial exact test at α = 0.05. n = 34 for Ft. Dix Strain and n = 27 for Bradenton Strain experiments.

In the untreated assays, bed bugs from the Ft. Dix and Bradenton strain consistently showed a preference for resting on the rougher P60 grit sanded side of all materials tested (*p <* 0.05; Figure 1). The Ft. Dix strain preferred to rest on the P60 grit sanded side of the assay 91%, 68%, and 91% of the time on copper, bass wood, and acrylic surfaces, respectively. With the Bradenton strain, 70% of the bed bugs chose to rest on the P60 grit sanded surface (*p =* 0.026; Figure 1). Mortality was not observed in any of these experiments lacking insecticide applications.

The Ft. Dix and Bradenton strain displayed remarkably different behaviors when exposed to the surfaces treated with Suspend SC or D-Force aerosol formulations (Table 1). Ft. Dix strain did not show a preference to either the P60 grit or P600 grit sanded acrylic surface when a Suspend SC formulation was applied to only the P60 grit side or when both the P60 grit and P600 grit sides were treated (*p =* 0.286; *p =* 0.575). A preference was also not found when both the P60 grit and P600 grit sides were treated with the D-Force aerosol formulation (*p =* 0.578). All individuals from these three treatment combinations died after three days post-assay. Ft. Dix bed bugs did show a preference for the P600 grit sanded side of the acrylic arena when D-Force aerosol formulation was applied to the P60 grit side (*p =* 0.014). Only 30% mortality was observed in individuals used in the assay with the P60 grit side treated with D-Force aerosol application.

**Table 1:** Roughness preferences and mortality of adult bed bugs given the choice between resting on a rougher P60 grit sanded half verses a P600 grit sanded half of a acrylic arena that is treated with a 0.06% deltamethrin formulation

Strain	TreatmentFormulation	Treatment Side	P60Grit Choice (%)	P600Grit Choice (%)	Mortality (%)
Ft. Dix	Suspend SC	P60Grit	57	43	100
	Suspend SC	Both	50	50	100
	D-Force Aerosol	P60Grit	27	73 **	30
	D-Force Aerosol	Both	50	50	100
Bradenton	Suspend SC	P60Grit	75 *	25	0
	Suspend SC	Both	76 *	24	0
	D-Force Aerosol	P60Grit	56	44	0
	D-Force Aerosol	Both	59	41	7

* Roughest surface was chosen more given a binomial exact test at α = 0.05; ** Roughest surface was chosen less given a binomial exact test at α = 0.05; No mortality was observed in control experiments.

Unlike the Ft. Dix strain, the Bradenton strain did show a preference for resting on the rougher P60 grit sanded acrylic side when Suspend SC formulation was applied to the P60 grit sanded side or both the P60 and P600 grit sides (*p =* 0.006; *p =* 0.007); however, no preference was observed when D-Force aerosol was applied (*p =* 0.351; *p =* 0.221). No mortality was observed in any of the treatments carried out except for 7% mortality when D-Force was applied to both the P60 and P600 grit sides of the acrylic arena.

## 4. Discussion

A consistent bed bug preference for resting on a rougher surface was found for all materials tested; however, we observed inconsistent behavioral responses in our pyrethroid application assays depending on the strain tested and which pyrethroid formulation was used.

Bed bugs displayed a preference for resting on rougher surface halves of metal, wood, and plastic surfaces tested and these results support previous studies from the early to mid 20th century examining roughness preferences [6,7,8]. A strong roughness preference and a thigmotactic behavior may aid bed bugs in selecting an ideal microclimate in which to harbor where they can enter a quiescent state until their next meal [8]. Authors Aboul-Nasr and Erakey [8] theorized that rough surfaced cracks and crevices likely have microclimates that are cool, hide individuals from direct sunlight, have low exposure to air currents, and have high humidity environments. All of these factors may aid the bed bug in reducing water loss during long quiescent states and protect bed bugs from detection by predators or their hosts [8].

Historically, switching a wooden bed frame to a metal bed frames was found to help in diminishing bed bug populations during control efforts. It was reasoned that metal bed frames had less cracks and crevices for bed bugs to hide in [14]. The low number of cracks and crevices in metal bed frames may not explain everything. We now know that bed bugs prefer to harbor in rougher surfaces and have difficulty climbing smooth surfaces [15]. These combining factors may explain some of the success seen in switching to metal bed frames during bed bug control efforts.

Investigations into the effects of pyrethroid applications were also conducted to examine if bed bug resting behavior would change during insecticide treatments. Applications of Suspend SC formulation (deltamethrin 0.06%) to the acrylic roughness preference arenas did not change the roughness preference of the Bradenton strain, but the Ft. Dix strain no longer displayed a roughness preference. It was clear that Bradenton did not elicit an avoidance response when contacting Suspend SC formulations and would still harbor on the preferred rougher half even if a Suspend SC formulation were present. Similar observations were observed in other studies examining the effects of a harborage preference factor against a possible repellent insecticide. In both bed bugs and German cockroaches, *Blattella germanica* (Blattodea: Blattellidae), avoidance was no longer observed when a harborage aggregation pheromone was present [13,16]. Other harborage preference factors such as negative phototaxis are not strong enough behaviors to mask the repellency effects of these chemicals [13]. Studies by another research group did not observe avoidance to Suspend SC formulations by Ft. Dix strain even when harborage characteristics were not taken into account [17]. Pyrethroids alone do not seem to cause an avoidance behavior in bed bugs when a dominant harborage characteristic is present.

The lack of a roughness preference observed in the Ft. Dix strain was likely from the individual’s nervous system becoming affected by deltamethrin, as suggested by the 100% mortality observed after three days. The Bradenton strain did not experience any mortality and likely possesses at least one or a combination of biochemical and physiological resistance mechanisms to pyrethroids. Further evidence of resistance in the Bradenton strain is supported by our unpublished results where direct applications of Hot Shot Bedbug and Flea Killer aerosol (Spectrum Brands, Middleton, WI, USA; Actives 0.025% prallethrin and 0.005% Gamma-Cyhalothrin) caused 37.5% mortality to adult Bradenton strain individuals but 100% mortality to Ft. Dix strain after 72 h post-treatment.

The Ft. Dix strain did not show a roughness preference when D-Force Aerosol formulation (deltamethrin 0.06%) was applied to both halves of the acrylic arena. This is likely because the bed bugs were under the effects of deltamethrin toxicity, as shown by the 100% mortality of all individuals tested. The behavior response of the Ft. Dix strain was different when D-Force aerosol formulation was applied only to the preferred rougher P60 sanded half of the arena. A strong avoidance response was observed to D-Force aerosol formulation, but because a similar response was not seen in the alternative Suspend SC formulation with the same concentration of deltamethrin, this avoidance behavior is likely caused by one or more of the other spray constituents. There could even be an interaction of multiple spray constituents causing the avoidance response. Spray constituents can include dispersants, emulsifiers, solvents, masking agents, synergists, repellents, and toxicants [16]. Aerosol formulations and other spray additives have been shown to cause repellency in other insects such as German cockroaches [16,18,19]. Avoidance behaviors observed in bed bugs from D-Force aerosol formulation is not likely due to a common factor found in all aerosols since Phantom aerosol (active ingredient: chlorfenapyr 0.5%) has been shown to be non-repellent [20]. Use of formulations with repellent properties could lead to control problems even when the bed bugs are susceptible to the active ingredient as seen in this study.

The lack of a roughness preference with the Bradenton strain when D-Force aerosol formulation was applied to the acrylic arena was unexpected. Because there is evidence that the Bradenton strain possesses at least some resistance to pyrethroids, one or more of the additive compounds within the aerosol formulation may be responsible for this change in behavior. The additive compound or compounds responsible may even be affecting the mechanoreceptors located on the antennae and other areas of the body directly [8,21].

## 5. Conclusions

This study has demonstrated the strong behavioral response of bed bugs to rough surfaces. Knowledge of the factors that affect bed bug harborage preference can aid pest control operators in their inspections. Although a rough surface preference is one of the many factors that bed bugs use to identify ideal harborage locations, these behaviors can be manipulated by chemical treatments. While it was clear that the pyrethroid itself was not a factor influencing the resting preference of bed bugs, additives in the formulation could influence bed bug behavior. Additives in formulations could play a primary role in failed control efforts even in the case of pyrethroid-susceptible bed bug populations. Further investigation is needed to examine the role some of these additives play in influencing bed bug resting behavior.
